# Odometer Velocity and Acceleration Estimation Based on Tracking Differentiator Filter for 3D-Reduced Inertial Sensor System

**DOI:** 10.3390/s19204501

**Published:** 2019-10-17

**Authors:** Qing Zhang, Lianwu Guan, Dexin Xu

**Affiliations:** College of Automation, Harbin Engineering University, Harbin 150001, China; zhq402@hrbeu.edu.cn (Q.Z.); xudexin@hrbeu.edu.cn (D.X.)

**Keywords:** land vehicles navigation, reduced inertial sensor system, velocity estimation, tracking differentiator filter, phase lag compensation

## Abstract

Velocity information from the odometer is the key information in a reduced inertial sensor system (RISS), and is prone to noise corruption. In order to improve the navigation accuracy and reliability of a 3D RISS, a method based on a tracking differentiator (TD) filter was proposed to track odometer velocity and acceleration. With the TD filter, an input signal and its differential signal are estimated fast and accurately to avoid the noise amplification that is brought by the conventional differential method. The TD filter does not depend on an object model, and has less computational complexity. Moreover, the filter phase lag is decreased by the prediction process with the differential signal of the TD filter. In this study, the numerical simulation experiments indicate that the TD filter can achieve a better performance on random noises and outliers than traditional numerical differentiation. The effectiveness of the TD filter on a 3D RISS is demonstrated using a group of offline data that were obtained from an actual vehicle experiment. We conclude that the TD filter can not only quickly and correctly filter velocity and estimate acceleration from the odometer velocity for a 3D RISS, but can also improve the reliability of the 3D RISS.

## 1. Introduction

Most current land vehicular navigation is highly dependent on the Global Positioning System (GPS). However, in urban canyons, tunnels, and other GPS-denied environments, GPS service may suffer from possible signal outages, jamming, and multi-path effects. To maintain positioning availability and accuracy in such cases, GPS is augmented with the inertial navigation system (INS). As a standalone approach, INS is inherently immune to external disturbances and is able to provide continuous navigation solutions with short-term accuracy [1]. Therefore, one of the common solutions for vehicular positioning during GPS outages is to augment GPS with INS [2,3].

For low-cost objectives, instead of integrating GPS with a full inertial measurement unit(IMU) containing three accelerometers and three gyroscopes, the reduced inertial sensor system (RISS) has gained more and more attention. Only one azimuth gyroscope and an odometer or wheel encoders is integrated with GPS, referred to as 2D RISS, to provide 2D positioning solutions in planar environments [1,4,5,6,7]. An integrated RISS/GPS module using a particle filter (PF) was proposed in [8] to provide 2D navigation solutions. A 3D RISS [2,9] composed of a 2D RISS and two horizontal accelerometers is another navigation solution suitable for all wheeled moving platforms, and could obtain the pitch and roll angles of a land vehicle. An enhanced version of PF called the Mixture PF was utilized in [10] to perform the tightly coupled integration of a 3D RISS with GPS. Currently, most studies on RISSs focus on the filters of RISS/GPS integration or on inertial sensor error correction techniques because of the low cost and low precision of the utilized inertial sensors [5,11,12].

An odometer is a completely autonomous device, whose measurement error divergence speed is slower than that of the inertial sensors. Therefore, the introduction of odometer measurement information in a pure inertial navigation system could slow down the divergence speed of INS error to a certain extent without decreasing the autonomous characteristics of the system.

In a 3D RISS, the derivative of velocity information from the odometers is used to calculate pitch and roll angles, and the velocity information from the odometer is used to calculate the position together with the inertial sensors. Additionally, during GPS outages, the velocity information from the odometers is also used for the RISS/GPS integrated navigation to correct the performance degradation with a Kalman filter (KF) [13,14,15]. However, in actual roadways, when the vehicle sideslips or jumps off the ground, the output of the odometer will not represent the actual velocity of the vehicle. Meanwhile, the output of the odometer is easily disturbed by random errors and some signal jumps because of the quantization errors and measurement noises of the wheel encoders. In these conditions, the error of the derivative of velocity information obtained in the classical numerical differential method is significantly boosted, which will decrease the navigation accuracy and reliability of the vehicle greatly. If the wrong velocity information is used in integrated RISS/GPS navigation, the error of the KF will diverge rapidly without restraint, especially during GPS outages. Moreover, the precision of the odometer for measuring the velocity of vehicles is low because of the cost of the odometers and the civilian vehicles overall. Therefore, it is necessary to improve the measurement accuracy and reliability of the odometer using error estimation technologies. Recently, many scholars have proposed various algorithms and approaches to improve this problem based on KFs [16,17]. However, KF and its improving algorithms need to build the object models to extract state information, but the actual velocity error model parameters cannot be measured accurately. In [18], a filter was presented that could achieve high-velocity estimation by fusing information from a magnetometer array with visual-inertial navigation systems (VINS), although the complexity and cost of the whole navigation system would be increased.

In engineering applications [19,20], it is common to encounter the requirements for extracting true signals or their derivatives from signals contaminated with noise. There have been various filtering methods proposed by researchers, such as the high-gain observer-based differentiator [21], adaptive filters [22], linear time-derivative trackers [23], robust exact differentiator [19,24], and so on. Actually, it is difficult to guarantee real-time performance because of the large computation complexity of these complicated filtering technology. Tracking Differentiator (TD), originally proposed by Han Wang [25], is mainly used to solve the problem of reasonably extracting continuous and differential signals from measurement signals with discontinuous or random noises. It overcomes the drawbacks of the classical differential algorithm and has a strong ability to suppress noises. Because of its rigid proof in mathematics and good tracking ability for applications in engineering, it has been widely used in various fields [26,27,28]. In [29], a method of extracting the velocity of a moving vehicle from the output signal of an odometer was introduced using TD to improve velocity precision. Compared with numerical differentiation, this method can not only restrain noise, but it also has a strong anti-interference ability, a simple algorithm, convenient parameter adjustment, and good filter effect. On the other hand, the phase lags and the differential output of the TD filter were not taken into account in this study [29].

Inspired by previous studies, this paper attempts to introduce the tracking-differentiator filter to extract proper velocities and their derivatives from odometers with noises, in order to improve the navigation accuracy and reliability of 3D RISSs in GPS-denied environments. In this 3D RISS, the original odometer velocity is filtered using a TD filter, the tracking signal output of the TD filter is used as the velocity, and the differential output of the TD filter is used as the acceleration. Using a group of offline data obtained from an actual vehicle experiment, the effectiveness of the TD filter on the 3D RISS was demonstrated in simulation experiments.

## 2. 3D RISS Mechanization and Error Analysis

### 2.1. 3D RISS Mechanization

**Figure 1:**
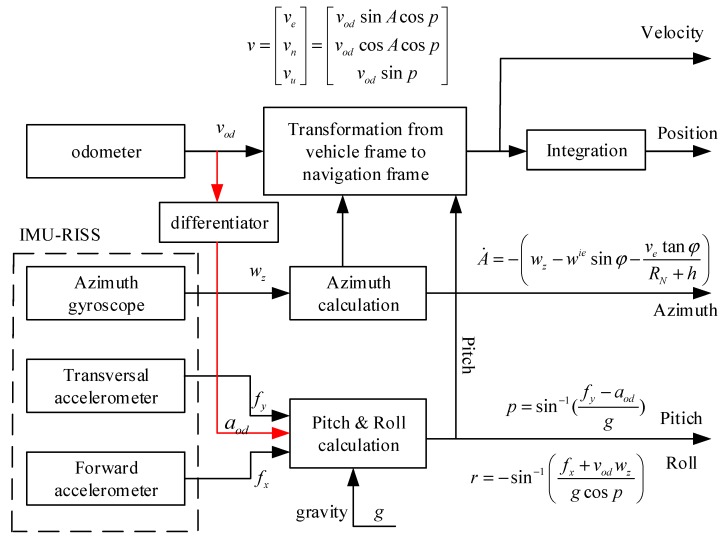
The schematic diagram of 3D RISS mechanization.

A 3D RISS is comprised of one azimuth gyroscope providing the azimuth angular rate change, two horizontal accelerometers for calculating the pitch and roll angles in a horizontal plan, and an odometer with a moving velocity in a near-horizontal plan [2,6,10,30]. The 3D RISS mechanization schematic diagram is shown in Figure 1.

During the 3D RISS mechanization process, the pitch angle of the vehicle is calculated by the forward accelerometer output information after error compensation. The pitch angle calculation formula is yielded by Equation (1):

(1) $$p=\sin^{-1}(\frac{f_{y}-a_{od}}{g}),$$

where *a_od_* is the acceleration of the vehicle in a near-horizontal plan, and *g* is the Earth’s gravity. 

*a_od_* is not directly measurable, and is obtained from the derivative of the odometer velocity *ν*_od_. Generally, *a_od_* at each time epoch can be calculated as

(2) $$a_{od}=\frac{v_{od}(k)-v_{od}(k-1)}{dt},$$

where *ν*_od_(*k*) refers to the *ν*_od_ at each time step, and *dt* is the sample epoch. 

After that, the roll angle of the dynamic vehicle is calculated by the transversal accelerometer information *f_x_*, the azimuth gyroscope measurement *w_z_*, and the odometer velocity information. Therefore, the roll angle calculation formula is yielded by Equation (3):

(3) $$r=-\sin^{-1}\left(\frac{f_{x}+v_{od}w_{z}}{g\cos p}\right),$$

Simultaneously, the azimuth can be derived by Equation (4).

(4) $$\dot{A}=-\left(w_{z}-w^{ie}\sin\varphi -\frac{v_{e}\tan\varphi }{R_{N}+h}\right),$$

After the attitude calculation, the 3D velocity in the local level frame (LLF) can be derived using Equation (5):

(5) $$v=\left[\begin{array}{c} v_{e}\\ v_{n}\\ v_{u} \end{array}\right]=\left[\begin{array}{c} v_{od}\sin A\cos p\\ v_{od}\cos A\cos p\\ v_{od}\sin p \end{array}\right],$$

Finally, the 3D position can be obtained by integration. The step-by-step computation of the 3D position is yield by

(6) $$\dot{h}=v_{u},\;\dot{\varphi }=\frac{v_{n}}{R+h},\;\dot{\lambda }=\frac{v_{e}}{(R+h)\cos\varphi },$$

### 2.2. Errors Analysis

#### 2.2.1. Attitude Errors Analysis

Differentiating Equations (1), (3), and (4), respectively, the attitude errors can be yield

(7) $$\delta p=\frac{\delta f_{y}-\delta a_{od}}{g\cos p}-\frac{(f_{y}-a_{od})\delta g}{g^{2}\cos p},$$

(8) $$\delta r=-\frac{\delta f_{x}+\delta v_{od}w_{z}+v_{od}\delta w_{z}}{g\cos p\cos r}+\frac{\left(f_{x}+v_{od}w_{z})(\delta g\cos p-g\delta p\sin p\right)}{{\left(g\cos p\right)}^{2}\cos r},$$

(9) $$\delta \dot{A}=-\left(\delta w_{z}-w^{ie}\delta \varphi \cos\varphi -\frac{\delta v_{e}\tan\varphi +v_{e}\delta \varphi \sec^{2}\varphi }{R_{N}+h}+\frac{v_{e}\tan\varphi \delta h}{{\left(R_{N}+h\right)}^{2}}\right),$$

From Equation (7), the pitch error is determined by the forward accelerometer measurement error *δf_y_*, the odometer’s forward accelerate error *δa_od_*, and the Earth’s gravity error *δg*. The roll error is determined by the transversal accelerometer measurement error *δf_x_*, the odometer measurement error *δv_od_*, the vertical gyroscope measurement error *δw_z_*, the pitch error *δp*, and also the Earth’s gravity error *δg*. Assuming that the Earth’s radius R is a relative large value, the azimuth error can be simplified as

(10) $$\delta \dot{A}=-\left(\delta w_{z}-w^{ie}\delta \phi \cos\phi \right),$$

Therefore, the azimuth error is mainly determined by the vertical gyro measurement error *δw_z_* and the latitude error *δφ*.

#### 2.2.2. Velocity Errors Analysis:

Differentiating Equation (5), the velocity errors can be yielded:

(11) $$\delta v_{e}=\delta v_{od}\sin A\cos p+\delta Av_{od}\cos A\cos p-\delta pv_{od}\sin A\sin p,$$

(12) $$\delta v_{n}=\delta v_{od}\cos A\cos p-\delta Av_{od}\sin A\cos p-\delta pv_{od}\cos A\sin p,$$

(13) $$\delta v_{u}=\delta v_{od}\sin p+\delta pv_{od}\cos p,$$

#### 2.2.3. Position Errors Analysis

Differentiating Equation (6), the position errors can be obtained:

(14) $$\delta \dot{h}=\delta v_{u},$$

(15) $$\delta \dot{\varphi }=\frac{\delta v_{n}}{R+h}-\frac{v_{n}\delta h}{{\left(R+h\right)}^{2}},$$

(16) $$\delta \dot{\lambda }=\frac{\delta v_{e}+\delta \varphi v_{e}\tan\varphi }{(R+h)\cos\varphi }-\frac{v_{e}\delta h}{{\left(R+h\right)}^{2}\cos\varphi },$$

In above analysis, the *ν*_od_ derived from the odometer is at least as important as the data from the gyroscope or accelerometers. The derivative *a_od_* of *ν*_od_ is used to calculate the pitch and roll angles. The *ν*_od_ is used to calculate the 3D position together with inertial sensors. Hence, in actual applications, it is of great significant to improve the measurement accuracy and reliability of the odometer velocity.

## 3. Tracking Differentiator Filter

### 3.1. TD Filter Principle

In classical control theory, the differentiator is built using a small time-constant inertial unit. The first-order derivate of the input signal U(s) can be obtained by following a linear, time-invariant, continuous-time dynamic system like Equation (17):

(17) $$Y=\frac{s}{Ts+1}U=\frac{1}{T}\left(1-\frac{1}{Ts+1}\right)U,$$

where Y(s) and U(s) are the output and input, respectively; *T* is the time constant; and *s* is the Laplace operator. In fact, when *T* is small enough, the inertia unit becomes an approximate time-delay unit. That is to say, 1/(*Ts* + 1) ≈ *e*^-*Ts*^. The inertia unit in Equation (17) could be treated as a time-delay unit with a small time constant. The inverse Laplace transform of Equation (17) is

(18) $$y(t)\approx \frac{1}{T}\left(u(t)-u(t-T)\right)\approx \dot{u}(t),$$

When signals u(t) are corrupted by noises, the noises are also amplified 1/*T* times; so, the differentiator obtained by the classical method is not suitable for most engineering applications. An improved differentiator of Equation (19) was proposed in [25].

(19) $$Y(s)=\frac{1}{T_{2}-T_{1}}\left(\frac{1}{T_{1}s+1}-\frac{1}{T_{2}s+1}\right)U(s),$$

The difference of the two inertia units is used as the differential to depress the noise amplification. In order to obtain the differential by the fastest dynamic part, a resulting control law that drives any initial state point to the origin in the minimum time is introduced to construct the noise-tolerant time optimal control (TOC)-based TD [27].

The double-integral system is defined as

(20) $$\left\{ \begin{array}{l} {\dot{x}}_{1}=x_{2}\\ {\dot{x}}_{2}=u \end{array}\right.,$$

where |*u*| ≤ *r*, and *r* is a constant constraint of the control input. It was proven in [24] that the resulting feedback control law that drives the state from any initial point to the origin in the shortest time is

(21) $$u=-R\cdot \mathrm{sat}(x_{1}-v+\frac{\left|x_{2}\right|x_{2}}{2R},\delta ),$$

where ***ν*** is the desired value for ***x***_1_.

(22) $$\mathrm{sat}(A,\delta )=\{\begin{array}{c} \mathrm{sign}(A),\left|A\right|>\delta \\ A/\delta ,\left|A\right|\leq \delta ,\delta >0 \end{array},$$

where sign(·) is the Sign function, so the TD filter is constructed by using

(23) $$\left\{ \begin{array}{l} {\dot{x}}_{1}=x_{2}\\ {\dot{x}}_{2}=-R\cdot \mathrm{sat}(x_{1}-v+\frac{\left|x_{2}\right|x_{2}}{2R},\delta ) \end{array}\right.,$$

where ***x***_1_ is the desired trajectory and ***x***_2_ is its derivative.

Via Euler’s method discretization, the discrete form of TD is given:

(24) $$\left\{ \begin{array}{l} fh=\mathrm{fhan}(x_{1}(k)-v(k),x_{2}(k),r,h_{0})\\ x_{1}(k+1)=x_{1}(k)+h\cdot x_{2}(k)\\ x_{2}(k+1)=x_{2}(k)+h\cdot fh \end{array}\right.,$$

where ***ν*** is the input signal, ***x***_1_ is the filter value of ***ν***, ***x***_2_ is the derivative of ***x***_1_, *r* is the tracking velocity of the TD filter, and *h* is the step size in simulation. The nonlinear switching function fhan (***x***_1_-***ν***, ***x***_2_, *r*, *h*_0_) is given by Equation (25):

(25) $$\begin{array}{l} fh=\mathrm{fhan}(x_{1}-v,x_{2},r,h_{0})\\ \left\{ \begin{array}{l} d=rh_{0}\\ d_{0}=h_{0}d\\ y=x_{1}-v+h_{0}x_{2}\\ a_{0}=\sqrt{d^{2}+8r\left|y\right|}\\ a=\left\{ \begin{array}{l} x_{2}+(a_{0}-d)\mathrm{sign}(y)/2,\left|y\right|>d_{0}\\ x_{2}+y/h_{0},\left|y\right|\leq d_{0} \end{array}\right.\\ \mathrm{fhan}=-\left\{ \begin{array}{l} r\mathrm{sign}(a),\left|a\right|>d\\ ra/d,\left|a\right|\leq d \end{array}\right. \end{array}\right. \end{array}$$

The state ***x***_1_ tracks the input signal ***ν*** in the maximum velocity *r* without oscillation due to the function of fhan. The error between ***x***_1_ and ***ν*** goes to zero. The larger the speed factor *r* is, the faster the signal tracks. However, the larger of the speed factor *r* is, the stronger the noise amplification is. Moreover, the noise will reduce by adjusting the filter parameter *h*_0_ (5~10 times of *h*). The larger the filter factor *h*_0_ is, the better the filtering effect is. However, the larger the filter factor *h*_0_ is, the greater the phase loss of the tracking signal. Therefore, in order to obtain a better filtering effect, coordinated adjustment of *r* and *h*_0_ is required.

Generally, there is some phase lag on the output results of filters. Because TD can give the derivative of the input signal, the phase lag of the TD filter could be compensated with the following equations:

(26) $$\left\{ \begin{array}{l} v_{1}(k)=v(k)+h_{1}\cdot x_{2}(k)\\ fh=\mathrm{fhan}(x_{1}(k)-v_{1}(k),x_{2}(k),r,h_{0}),\\ x_{1}(k+1)=x_{1}(k)+h\cdot x_{2}(k),\\ x_{2}(k+1)=x_{2}(k)+h\cdot fh, \end{array}\right.,$$

where ***ν***_1_ is the new input signal composed of the original input signal ***ν***, *h*_1_ is the forecast time of ***ν*** with ***x***_2_, and ***x***_1_ is the filter value of ***ν***_1_. The forecast time *h*_1_ is usually 1~1.5 times of *h*_0_.

### 3.2. TD Filter Simulation Examples

#### 3.1.1. Phase Compensation for Signal Filtering

**Figure 2:**
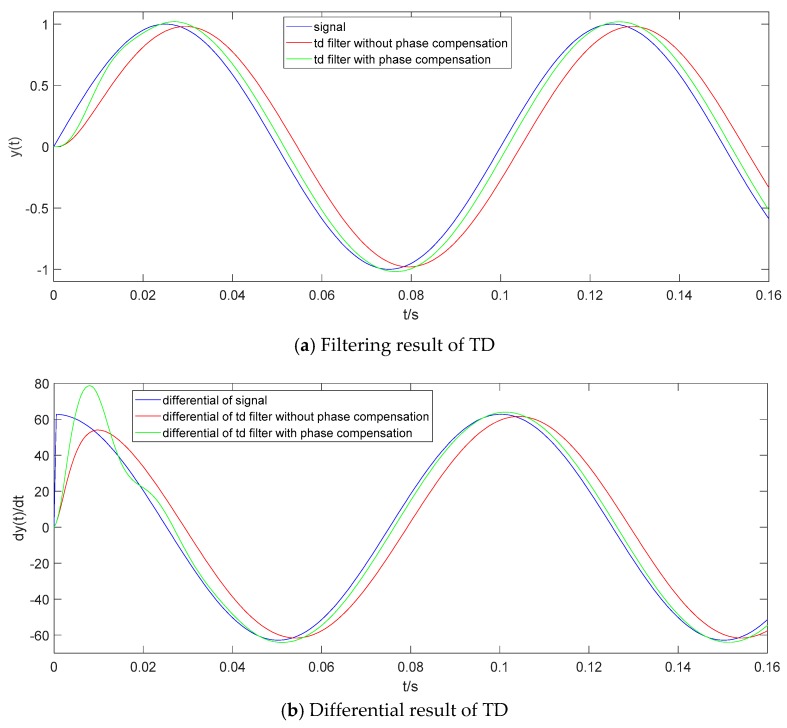
Filtering result and differential result of TD.

Let y(t) = sin(20πt) be an original input signal. A TD filter is set with the given design parameters: *h* = 0.005 s, *h*_0_ = 5 h, r = 30,000, and *h*_1_ = 1.2 *h*_0_. The original input signal y(t), the filtering result of TD without phase compensation and the filtering result of TD with phase compensation are shown in Figure 2a. As Figure 2a shows, the phase lag of the TD filter is obviously reduced by the phase compensation. The numerical differential result of y(t), the differential result of TD without phase compensation and the differential result of TD with phase compensation are shown in Figure 2b. As Figure 2b shows, after half a cycle of y(t), the differential result of TD with phase compensation can track the numerical differential result of y(t) very well. So, in the following part of this paper, TD refers in particular to TD with phase compensation.

#### 3.1.2. Noise Reduction

**Figure 3:**
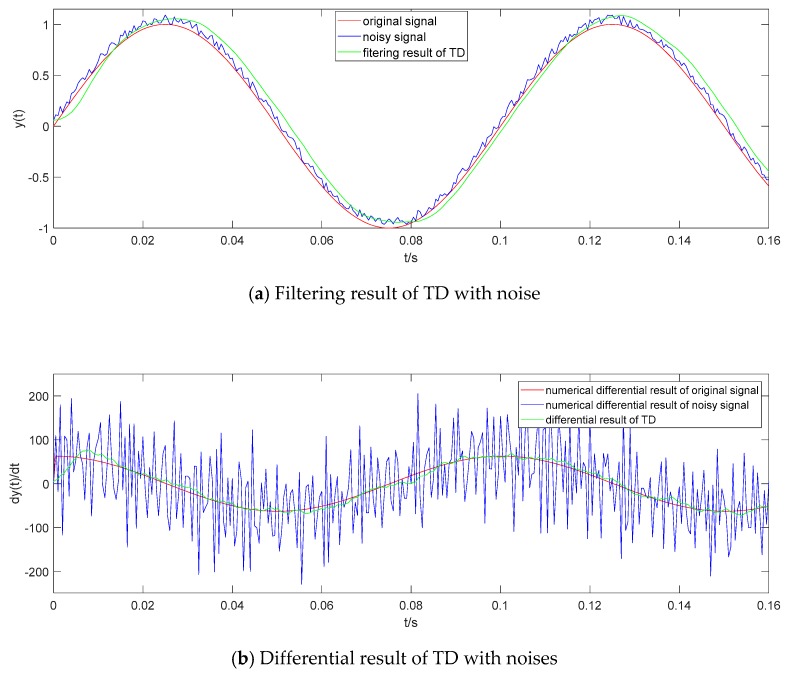
Filtering result and differential result of TD with noise.

A random noise with uniform distribution in [0,0.1] is added to y(t). The given design parameters of the above TD filter remain unchanged. The original input signal y(t), the noisy signal and the filtering result of TD are shown in Figure 3a. TD can extract the original signal from the noisy signal with a small phase lag. The numerical differential result of original signal and noisy signal, and the differential result of TD are shown in Figure 3b. Compared to the numerical differential result of the original signal, the differential result of TD is obviously more accurate than the numerical differential result of the noisy signal.

#### 3.1.3. Outliers Exclusion

**Figure 4:**
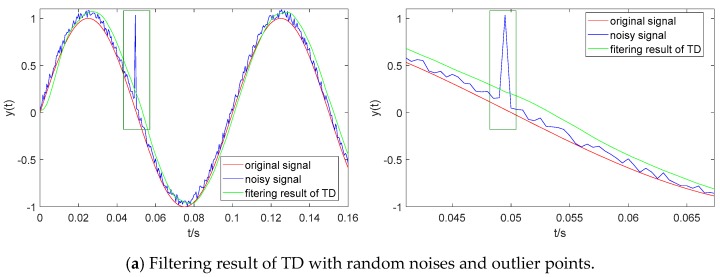

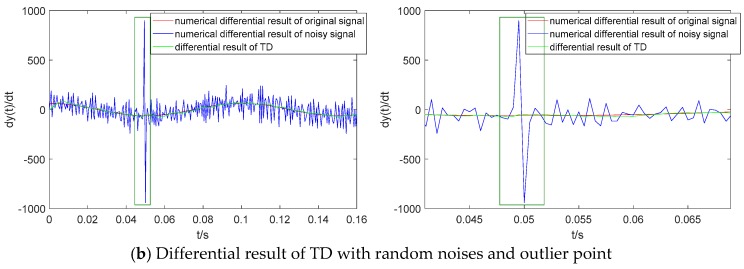
Filtering result and differential result of TD with random noises and outlier point.

Here, a hypothetical outlier point is magnified 10x from the noisy signal referred to in Section 3.1.2. In addition, the given design parameters of the above TD filter remain unchanged. The original input signal y(t), the noisy signal and the filtering result of TD are shown in Figure 4a. TD cannot only extract the original signal from the noisy signal with a small phase lag, but is also hardly affected by the outlier point. The numerical differential result of the original signal and noisy signal, and the differential result of TD are shown in Figure 3b. The outlier point leads to the remarkable error of the numerical differential result of the noisy signal. However, there is almost no effect caused by the outlier point on the differential result of TD.

As Section 3 demonstrated, TD achieves a better performance on random noises and outliers than traditional numerical differentiation. Using the odometer velocity data of a 3D RISS as the input signal of a TD filter, the filtered value of the odometer velocity data can be obtained from ***x***_1_ of this TD with noise reduction and outlier exclusion, and the acceleration data can also be obtained from ***x***_2_ of this TD filter rather than being calculated using the numerical differential method.

## 4. Simulation Experiments

In this section, a group of offline data prepared for a study of RISSs by the Navigation Instrumentation Research Group in Royal Military College of Canada is used to demonstrate the effect of velocity and acceleration estimation through an actual vehicular experiment based on TD. The 3D RISS mechanization is constructed from a low-grade Xbow IMU with the odometer output at 10 Hz update rates, and high-end Novatel SPAN IMU mechanization results are used as the reference. The odometer velocity (preprocessed by an offline wavelet filter) of this group is used as the original odometer velocity. The original velocity is filtered through a TD filter set with the given design parameters: *h* = 0.1 s, *h*_0_ = 5 h, r = 20,000, and *h*_1_ = 1.4 *h*_0_. In the 3D RISS with the original odometer velocity filtered by TD, the ***x***_1_ takes the place of the original velocity *ν*_od_ and the ***x***_2_ takes the place of *a_od_* calculated by Equation (2). The errors in attitude, velocity, position, and 2D trajectory will be plotted for comparison. Finally, error analysis will be discussed.

### 4.1. Validation of a 3D RISS with Velocity Filtered by TD 

**Figure 5:**
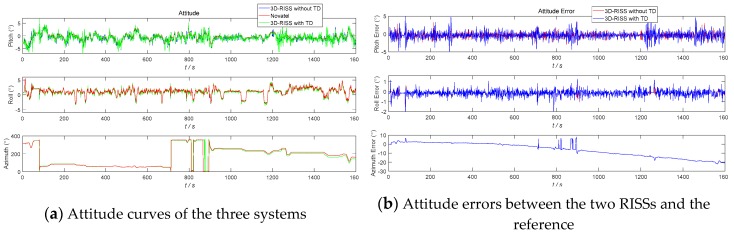
RISS attitude and attitude errors with the original odometer velocity filtered by TD.

**Figure 6:**
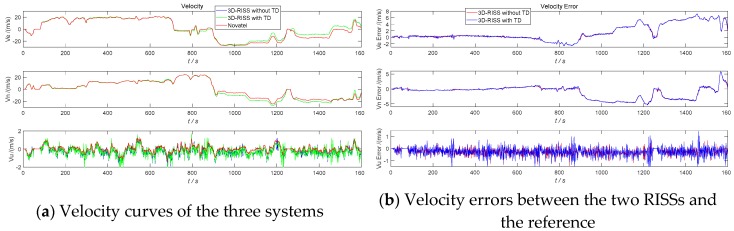
RISS velocity and velocity errors with the original odometer velocity filtered by TD.

**Figure 7:**
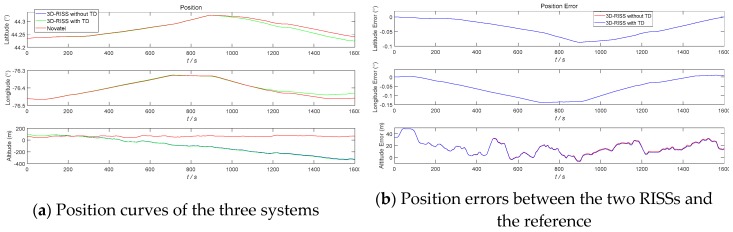
RISS position and position errors with the original odometer velocity filtered by TD.

**Figure 8:**
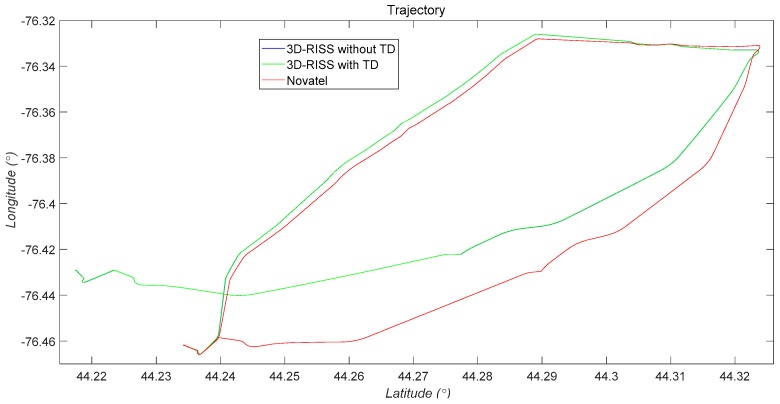
2D trajectories comparison between the two RISSs and the reference.

The three navigation simulation results of the 3D RISS with the original odometer velocity without a TD filter, the 3D RISS with the original odometer velocity filtered by TD, and the Novatel IMU are compared in Figure 5, Figure 6, Figure 7 and Figure 8. In Figure 5, Figure 6, Figure 7 and Figure 8, “3D-RISS without TD” refers to the simulation results of the 3D RISS with the original odometer velocity without a TD filter, “3D-RISS with TD” refers to the simulation results of the 3D RISS with the original odometer velocity filtered by TD, and “Novatel” refers to the simulation results of the Novatel IMU. The standard deviations of the main navigation errors between the two 3D RISSs and Novatel IMU are listed in Table 1.

Figure 5a depicts a comparison of the tri-axial attitude curves. The two attitude errors between the two types of 3D RISSs and the Novatel IMU are plotted in Figure 5b. The pitch and roll data of the two types of 3D RISSs have a similar variation tendency with the Novatel attitude reference and no divergence trend. However, the azimuth error curves are divergent with time, which is mainly caused by the vertical gyro measurement error *δω_z_*. According to the 3D RISS mechanization, the azimuth error divergence cannot be corrected with the odometer velocity. The standard deviations of the pitch and roll errors between the 3D RISS with the original odometer velocity and the Novatel IMU are 0.88° and 0.32°, respectively. The standard deviations of the pitch and roll errors between the 3D RISS with the original odometer velocity filtered by TD and the Novatel IMU are 1.16° and 0.36°, respectively. Because of the filter phase lag, the horizontal attitude error with the TD filter is a little bigger. 

Figure 6a depicts a comparison of the tri-axial velocity curves. The two velocity errors between the two types of 3D RISSs and the Novatel IMU are plotted in Figure 6b. As the azimuth error increases, the east velocity V_E_ error and the north velocity V_N_ error of the two RISSs also increases. While the pitch is not divergent, neither is the error of the vertical velocity V_U_. The results of the V_E_, V_N_, and V_U_ of the two 3D RISS do not coincide with each other. The standard deviations of the V_E_, V_N_, and V_U_ errors between the 3D RISS with the original odometer velocity and the Novatel IMU are 2.40 m/s, 2.13 m/s, and 0.28m/s, respectively. The standard deviations of the V_E_, V_N_, and V_U_ errors between the 3D RISS with the original odometer velocity filtered by TD and the Novatel SPAN IMU are 2.41 m/s, 2.13 m/s and 0.35 m/s, respectively.

Figure 7a depicts a comparison of tri-axial position curves. The two position errors between the two types of 3D RISSs and the Novatel SPAN IMU are plotted in Figure 7b. As the horizontal velocity error increases, the latitude and longitude errors of the two RISSs also increase. The two altitude errors of the two 3D RISSs have the same divergence tendency from the beginning of the 3D RISS mechanization, which is because of the open loop characteristic of the 3D RISS mechanization in the vertical channel. Similar to the velocity results, the positions of the two 3D RISSs coincide with each other as well. The standard deviations of latitude and longitude errors between the 3D RISS with the original odometer velocity and the Novatel SPAN IMU are 0.029107° and 0.052164°, respectively. The standard deviations of latitude and longitude errors between the 3D RISS with the original odometer velocity filtered by TD and the Novatel SPAN IMU are 0.029109° and 0.052155°, respectively. The position results of the 3D RISS with the original odometer velocity filtered by TD are slightly better than those of the 3D RISS with the original odometer velocity.

The three 2D trajectory simulation results of the 3D RISS with the original odometer velocity, the 3D RISS with the original odometer velocity filtered by TD, and the Novatel IMU are compared in Figure 8. The two trajectory errors between the two types of 3D RISSs and the Novatel IMU are almost identical.

As Figure 5, Figure 6, Figure 7 and Figure 8 and Table 1 show, the two navigation errors of the 3D RISS with the original odometer velocity and the 3D RISS with the original odometer velocity filtered by TD occur at the same level. Using the original odometer velocity, TD could quickly and correctly filter the velocity and estimate the acceleration for a 3D RISS.

### 4.2. Anti-Interference Ability of a 3D RISS with Velocity Filtered by TD

**Figure 9:**
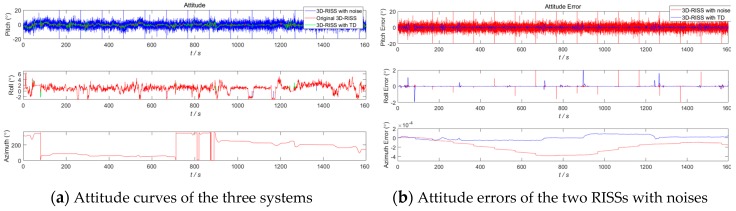
RISS attitude and attitude errors with a noisy odometer velocity filtered by TD.

**Figure 10:**
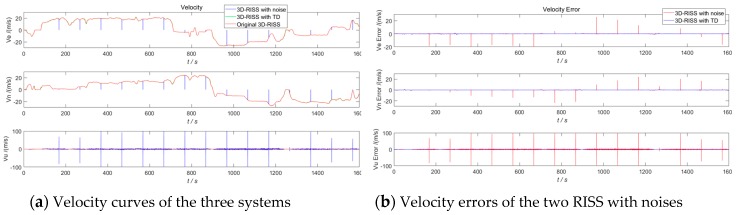
RISS velocity and velocity errors with a noisy odometer velocity filtered by TD.

**Figure 11:**
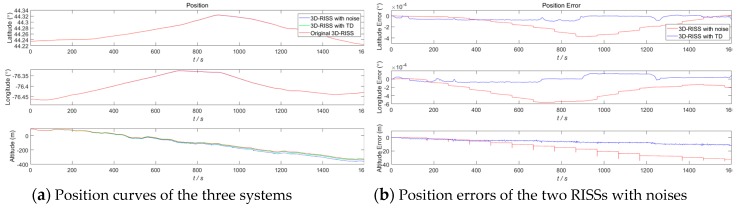
RISS position and position errors with a noisy odometer velocity filtered by TD.

**Figure 12:**
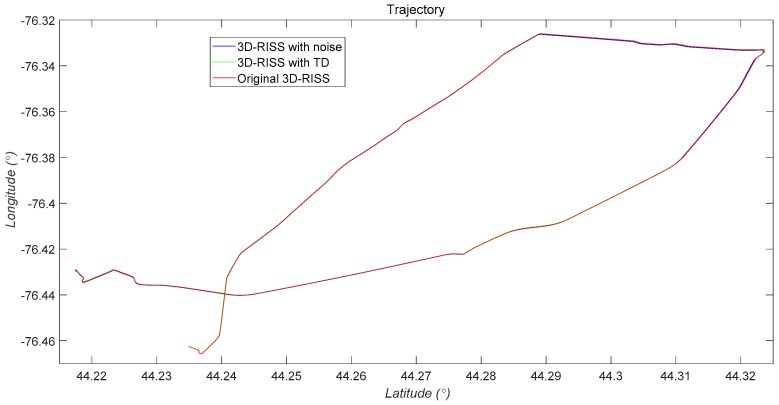
2D trajectory comparison between the two RISSs with noises and the reference.

In order to demonstrate the anti-interference effect of TD on a 3D RISS, a white Gaussian noise (with 0.002 variance and a 0 mean value) and a hypothetical outlier point (2 times amplitude and 100 s interval) are added to the original odometer velocity. The white Gaussian noise model is in accordance with our Kalman filter for the RISS/GPS integration, and the hypothetical outlier point is to simulate the odometer velocity error caused by vehicle sideslips or jumps off the ground. The given design parameters of the above TD filter remain unchanged. The results in Section 4.1 of the 3D RISS with the original odometer velocity are used as the reference. The three navigation simulation results of the 3D RISS with a noisy odometer velocity without a TD filter, the 3D RISS with a noisy odometer velocity filtered by TD, and the 3D RISS with the original odometer velocity are compared in Figure 9, Figure 10, Figure 11 and Figure 12. In Figure 9, Figure 10, Figure 11 and Figure 12, “3D RISS with noise” refers to the simulation results of the 3D RISS with a noisy odometer velocity without a TD filter, and “3D RISS with TD” refers to the simulation results of the 3D RISS with a noisy odometer velocity filtered by TD, and “Original 3D RISS” refers to the simulation results of the 3D RISS with the original odometer velocity. The standard deviations of the navigation errors between the two 3D RISSs with a noisy odometer velocity and the reference are listed in Table 2.

Figure 9a depicts a comparison of the tri-axial attitude curves. The two attitude errors between the two 3D RISSs with noisy odometer velocities and the reference are plotted in Figure 9b. Because the *a_od_* is in the calculation of the pitch, the pitch errors are most obviously affected in the comparison of attitude curves. The max pitch error between the 3D RISS with the original odometer velocity and the 3D RISS with a noisy odometer velocity reaches 91°. However, the max pitch error between the 3D RISS with the original odometer velocity and the 3D RISS with a noisy odometer velocity filtered by TD is 11°. The standard deviations of the pitch and roll errors between the 3D RISS with the original odometer velocity and the 3D RISS with a noisy odometer velocity are 10.61° and 0.065°, respectively. The standard deviations of the pitch and roll errors between the 3D RISS with the original odometer velocity and the 3D RISS with a noisy odometer velocity filtered by TD are 1.75° and 0.18°, respectively. 

Figure 10a depicts a comparison of the tri-axial velocity curves. The two velocity errors between the two 3D RISSs with noisy odometer velocities and the reference are plotted in Figure 10b. The standard deviations of the V_E_, V_N_, and V_U_ errors between the 3D RISS with the original odometer velocity and the 3D RISS with a noisy odometer velocity are 24.95 m/s, 24.19 m/s, and 34.79 m/s, respectively. The standard deviations of the V_E_, V_N_, and V_U_ errors between the 3D RISS with the original odometer velocity and the 3D RISS with a noisy odometer velocity filtered by TD are 0.26 m/s, 0.25 m/s, and 0.64 m/s, respectively. Due to the excellent filtering performance of TD in velocity estimation, the velocity results of the 3D RISS with a noisy odometer velocity filtered by TD is hardly influenced by the hypothetical noises.

Figure 11a depicts a comparison of the tri-axial position curves. The two position errors between the two 3D RISSs with noisy odometer velocities and the reference are plotted in Figure 11b. The standard deviations of latitude and longitude errors between the 3D RISS with the original odometer velocity and the 3D RISS with a noisy odometer velocity are 0.0021° and 0.0029°, respectively. The standard deviations of latitude and longitude errors between the 3D RISS with a noisy odometer velocity filtered by TD and the 3D RISS with the original odometer velocity are 0.000039° and 0.000077°, respectively. Similarly, the differences of the above two velocity errors are reflected in the two position errors. The three 2D trajectory simulation results of the three types of 3D RISS are compared in Figure 12. 

As Figure 9, Figure 10, Figure 11 and Figure 12 and Table 2 show, with the hypothetical significant noises introduced into the original odometer velocity, the divergence speed of navigation errors of the 3D RISS with odometer velocity filtered by a TD filter is much slower than the pure 3D RISS. Thus, the reliability of a 3D RISS is obviously improved by a TD filter used for estimating velocity and acceleration. Additionally, the velocity filtered by TD is favorable to reduce the error divergence risk of the integrated navigation Kalman filter.

## 5. Conclusions

Compared to a lot of studies on RISSs that have focused on filters of RISS/GPS integration or on the error correction of inertial sensors, there have seldom been studies on odometer velocity for a 3D RISS. However, in 3D RISS mechanization and error analysis, velocity information and its derivatives are at least as important as the information from the other inertial sensors of the RISS. Velocity information from an odometer is prone to noise corruption, which further leads to the noise amplification of acceleration information in a conventional differential method. This paper has presented a solution for odometer velocity and acceleration estimation using a 3D RISS based on a TD filter. 

A TD filter does not depend on an object model and has less computation. With a TD filter, an input signal and its differential signal are estimated fast and accurately. Additionally, using the differential signal output by the TD filter, the filter phase lag can be decreased with the prediction method. As Section 3 demonstrated, TD achieves better performance on random noises and outliers than traditional numerical differentiation.

Using a group of offline data obtained from an actual vehicle experiment, the effectiveness of a TD filter on a 3D RISS was demonstrated through simulation experiments. With the odometer velocity data of a 3D RISS as the input signal of a TD filter, the filtered value with noise reduction and the exclusion of outlying odometer velocity data can be obtained from tracking the signal output from this TD, and the acceleration data can be obtained from the derivative output of this TD also, rather than calculated using the numerical differential method. As the results show, a TD filter could not only correctly and quickly filter the velocity and estimate the acceleration from the odometer velocity using a 3D RISS, but could also improve the reliability of a 3D RISS. 

The future work will be to transform the TD filter algorithm into a navigational computer program of an actual 3D RISS.

## Figures and Tables

**Table 1:** Standard deviations of main navigation errors between the two 3D RISSs and the Novatel IMU.

Error	3D RISS with Original Odometer Velocity	3D RISS with Original Odometer Velocity Filtered by TD
pitch	0.88°	1.16°
roll	0.32°	0.36°
V_E_	2.40 m/s	2.41 m/s
V_N_	2.13 m/s	2.13 m/s
V_U_	0.28 m/s	0.35 m/s
latitude	0.029107°	0.029109°
longitude	0.052164°	0.052155°

**Table 2:** Deviations of the main navigation errors between the two 3D RISSs with noisy odometer velocities and the reference.

Error	3D RISS with Noisy Odometer Velocity	3D RISS with Noisy Odometer Velocity Filtered by TD
pitch	10.61°	1.75°
roll	0.065°	0.18°
V_E_	24.95 m/s	0.26 m/s
V_N_	24.19 m/s	0.25 m/s
V_U_	34.79 m/s	0.64 m/s
latitude	0.0021°	0.000039°
longitude	0.0029°	0.000077°
